# Taxonomical modeling and classification in space hardware failure reporting

**DOI:** 10.1038/s41598-026-36813-7

**Published:** 2026-01-21

**Authors:** Daniel Palacios, Terry R. Hill

**Affiliations:** 1 Jan and Dan Duncan Neurological Research Institute at Texas Children’s Hospital, Houston, TX USA; 2 Graduate Program of Quantitative & Computational Biosciences, Baylor College of Medicine, Houston, TX USA; 3 NASA, Engineering Processes and Methods Branch, Johnson Space Center, Houston, TX USA

**Keywords:** Computational science, Aerospace engineering

## Abstract

NASA Johnson Space Center has collected more than 54,000 space hardware failure reports. Obtaining engineering processes trends or root cause analysis by manual inspection is impractical. Fortunately, novel data science tools in Machine Learning and Natural Language Processing (NLP) can be utilized to perform text mining and knowledge extraction. In NLP the use of taxonomies (classification trees) are key to the structuring of text data, extracting knowledge and important concepts from documents, and facilitating the identification of correlations and trends within the data set. Usually, these taxonomies and text structures live in the heads of experts in their specific field. However, when an expert is not available, taxonomies and ontologies are not found in data bases, or the field of study is too broad, this approach can enable and provide structure to the text content of a record set. In this paper an automated taxonomical model is presented by the combination of Latent Dirichlet Allocation (LDA) algorithms and Bidirectional Encoder Representations from Transformers (BERT). Additionally, the limitations and outcomes of causal relationship rule mining models, commercial tools, and deep neural networks are also discussed.

## Introduction

**Fig. 1:**
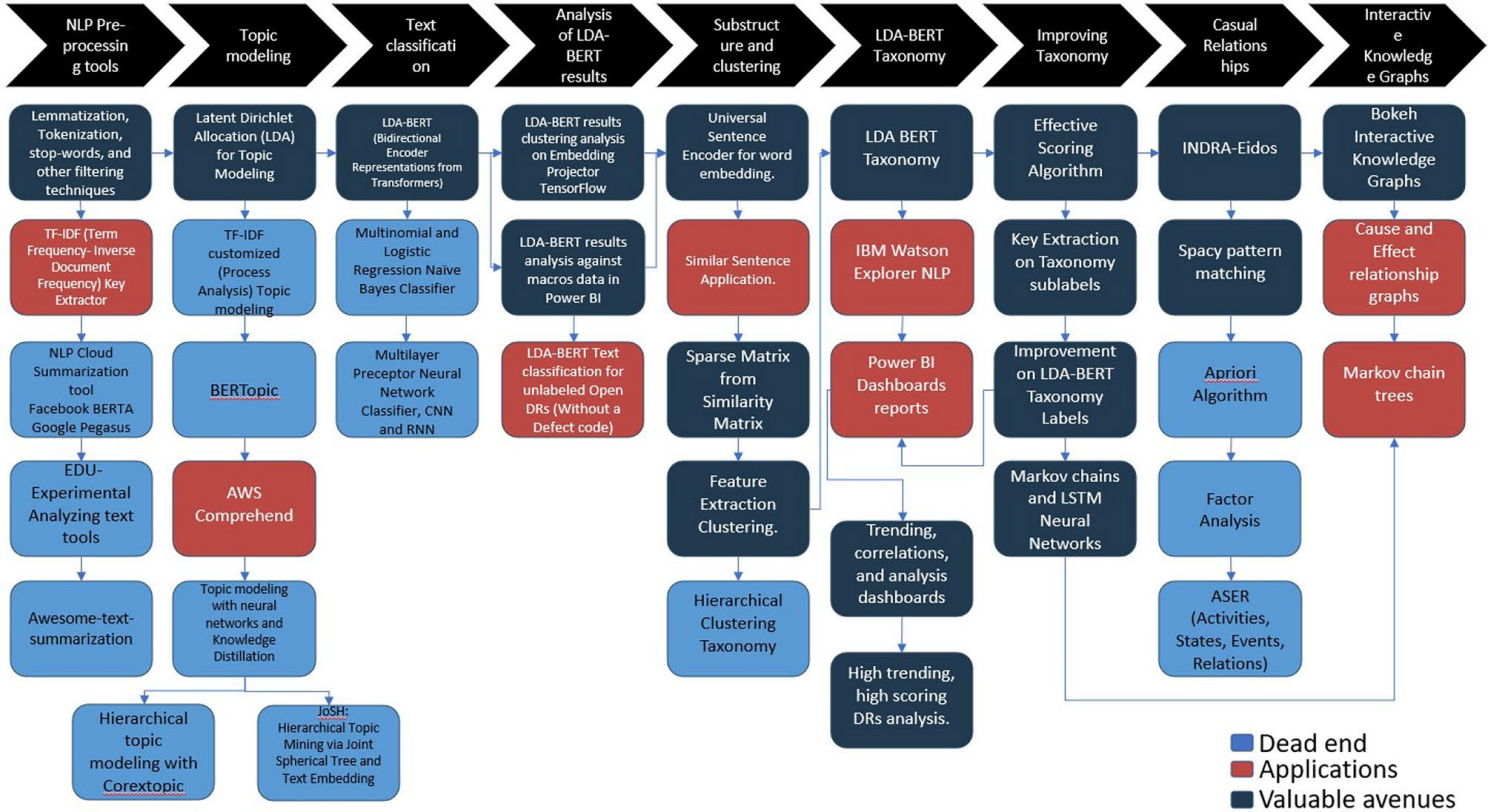
Natural Language Processing Space Hardware Discrepancy Reports Research Map. Each square represent a machine learning technique, NLP concept, commercial tool, or application developed tried. There are placed in non-chronological order and grouped by the each step of the text mining process.

Since the time when NASA’s Lyndon B. Johnson Space Center truly “went digital” with common usage of data systems (databases), most of the information has been captured and managed in digitized versions of the paper-based document predecessors. This has made leveraging the data a very big challenge because due to budgetary constraints there has been little enterprise-level effort put into how to use this data and the information, and correlations that may reside within, to inform the Engineering Directorate (mail code EA) as to how to better execute our responsibilities and improve our deliverables in support of human space flight. Starting in 2019 EA underwent significant effort to revamp the directorate tools and processes to help ensure successful missions to the Moon in the coming years. An important part of this effort is focused on finding ways to best leverage data and increase data analytical capabilities. From a business rhythm perspective, this area is relatively new to the Engineering directorate when it comes to analyzing the data associated with directorate processes designed to ensure consistent and expected outcomes of development and delivery of certified flight hardware. There has been some rudimentary analysis to date of hardware Discrepancy Report (DR) data utilizing MS Excel to identify the leading DR codes associated with EA-developed and certified hardware over the last two decades. However, additional correlations and root cause analysis is necessary to establish common causes and trending, and specific correlations between hardware processes to provide the information required to correctly inform corrective actions design to reduce the frequency of DRs in areas which drive program and project cost, schedule, and safety. After some initial analysis of the DRs, most of the information to identify root causes and processes correlations were discovered to be hidden inside the free-form text of the records. The number of records corresponds to more than 54,000 documents. Thus, manual root cause identification was not deemed economic use of labor. Due to the large number of records and unstructured nature, there was no clear systematic way of generating knowledge from them. Careful planning took placed to explore the different NLP open source and commercial available tools to test them and compare their results. A map illustrating the viable tools, researched dead ends, and applications developed is shown in Fig. 1.

NLP has evolved significantly in recent years, leading to advancements in text classification. Text classification, a fundamental task in NLP, involves categorizing text into predefined classes, making it a vital tool for information retrieval, sentiment analysis, and spam detection. The initial approaches to text classification were largely based on rule-based systems and traditional machine learning techniques, such as Naive Bayes, Support Vector Machines (SVMs), and Decision Trees. These methods relied heavily on hand-crafted features and linguistic analysis^1,2^. The advent of deep learning brought significant improvements in text classification. Convolutional Neural Networks (CNNs) and Recurrent Neural Networks (RNNs), including Long Short-Term Memory (LSTM) networks, have been extensively used for their ability to capture semantic and syntactic dependencies in text^3^. The introduction of the transformer architecture, particularly models like BERT (Bidirectional Encoder Representations from Transformers)^4^, revolutionized NLP. These models, pre-trained on vast text corpora, have set new benchmarks in text classification by effectively capturing contextual information.

Topic modeling is an NLP technique used to uncover hidden thematic structures in large text corpora. It is essential for summarizing, understanding, and organizing large datasets. Latent Dirichlet Allocation (LDA)^5^ is a generative probabilistic model that identifies topics based on word co-occurrence patterns. LDA has been a foundational approach in topic modeling, inspiring numerous subsequent models and applications. Various extensions to LDA have been proposed to enhance its flexibility and accuracy, such as the Hierarchical Dirichlet Process (HDP)^6^, and dynamic topic models to capture topic evolution over time^7^. The integration of neural networks into topic modeling has opened new avenues for improving topic coherence and interpretability. Models like the Neural Variational Inference for Text Processing (NVITP)^8^ combine deep learning with traditional topic modeling techniques. Recent works have focused on incorporating contextual embeddings from transformer models into topic modeling, enhancing the ability to capture nuanced and context-specific topics^9^.

Machine Learning models are usually built to work with numerical data; however, text can be transformed into numbers by a word-vector representation^10^ or by a Term Frequency and Inverse Document Frequency (TF-IDF) matrix^11^. In order for this numerical representation to be more accurate, with associated higher-performing models, some pre-processing techniques are needed. First, unwanted special characters are deleted (e.g. & % #) and digits are filtered because these characters don’t provide useful information for the analysis of space hardware. Note that in Sentiment Analysis, this ‘unwanted special characters’ might be useful since punctuation is important to weight human sentiment (e.g. This cake is good!). Then, sentences are split into single words which is known as Tokenization. Furthermore, there are sometimes attached words in the text, it is useful to split them (e.g. Anchorseed $\rightarrow$ anchor seed). Although capitalization of words is important grammatically, it does not carry important information with regard to the count or TF-IDF of words in a text. To avoid putting the same word in different bins (e.g. Run and run), the next step is to normalize all tokens (single words) to lower case. In the sake of having a simpler text, additional strategies are implemented such as Lemmatization and Stemming. These two techniques transform a word into its more simple constituent, e.g. running $\rightarrow$ run, leaked $\rightarrow$ leak. Note that usually ‘leaked’ and ‘leak’ would have increase the size to the resulting matrices, however after Lemmatization and Stemming the number of dimensions is reduced.

When analyzing a set of document texts it is often resourceful to run a topic modeling labeling technique with the objective of extracting text structure from a record. The generation of labels for each document transforms a unstructured text to a structured one by providing a way to classify them. With a classification generated it is possible to analyze individual groups of records. Several models were tested such as Customized Topic Modeling with TF-IDF key extractor and BERTopic (See Methods "Other topic modeling and text classification tools tested"); however, the best topic modeling technique found was the LDA approach^12^ for this use-case after several accuracy and validation measures. Other models performed poorly in accuracy or failed to yield in optimal number of labels across different parameters. Specifically, BERTopic gave same scores for a range of number of topics which made impossible the determination of the optimal number of topics within a DR defect code. The customized Topic modeling didn’t perform with a stable accuracy across the different DR defect codes, also this model was compared directly to LDA, LDA had higher accuracies across different text classification models. Amazon Web Services (AWS) Comprehend topic modeling feature was also tested, although accuracy wasn’t calculated explicitly, a careful inspection of the labels themselves denote that LDA labels were more informative for our use case. One of the main advantages of LDA over the other approaches tried was the ability to take into account the overlapping of topics in a single document, which is more representative of real-world incident reports and documentation, rather than a specific, single topic per document. In our research, we aim to assess the real-world applicability of text classification and topic modeling techniques using actual data. To achieve this, we methodically apply a variety of recognized methods to a unique dataset comprising NASA’s space failure records.

## Results

### LDA-BERT automated taxonomy identified key high trending engineering processes failures in discrepancy reports

Natural Language Processing techniques for Topic Modeling and Text Classification for DR text mining were developed and investigated. A road map displaying different models explored is shown in Fig. 1. A set of ‘process-labels’ or topics were created for each of the DR codes to identify, classify, and analyze the key phrases and concepts within the text of the DRs. Visualization and analysis software, Power BI, and embedding projector from TensorFlow were explored. Some applications were also developed: similar sentence query and text classification for unlabeled DRs (records without a defect code). With the creation of a Taxonomy from LDA-BERT model, the documents can be analyzed easier in subgroups with filtered text (clean text documents after NLP-preprocessing techniques). The JSC team reached out to several teams across NASA including the Langley Research Center (LARC) to discuss commercial tools available within the Agency such as AWS Comprehend, Google Cloud Platform NLP services, and Watson Explorer.

**Fig. 2:**
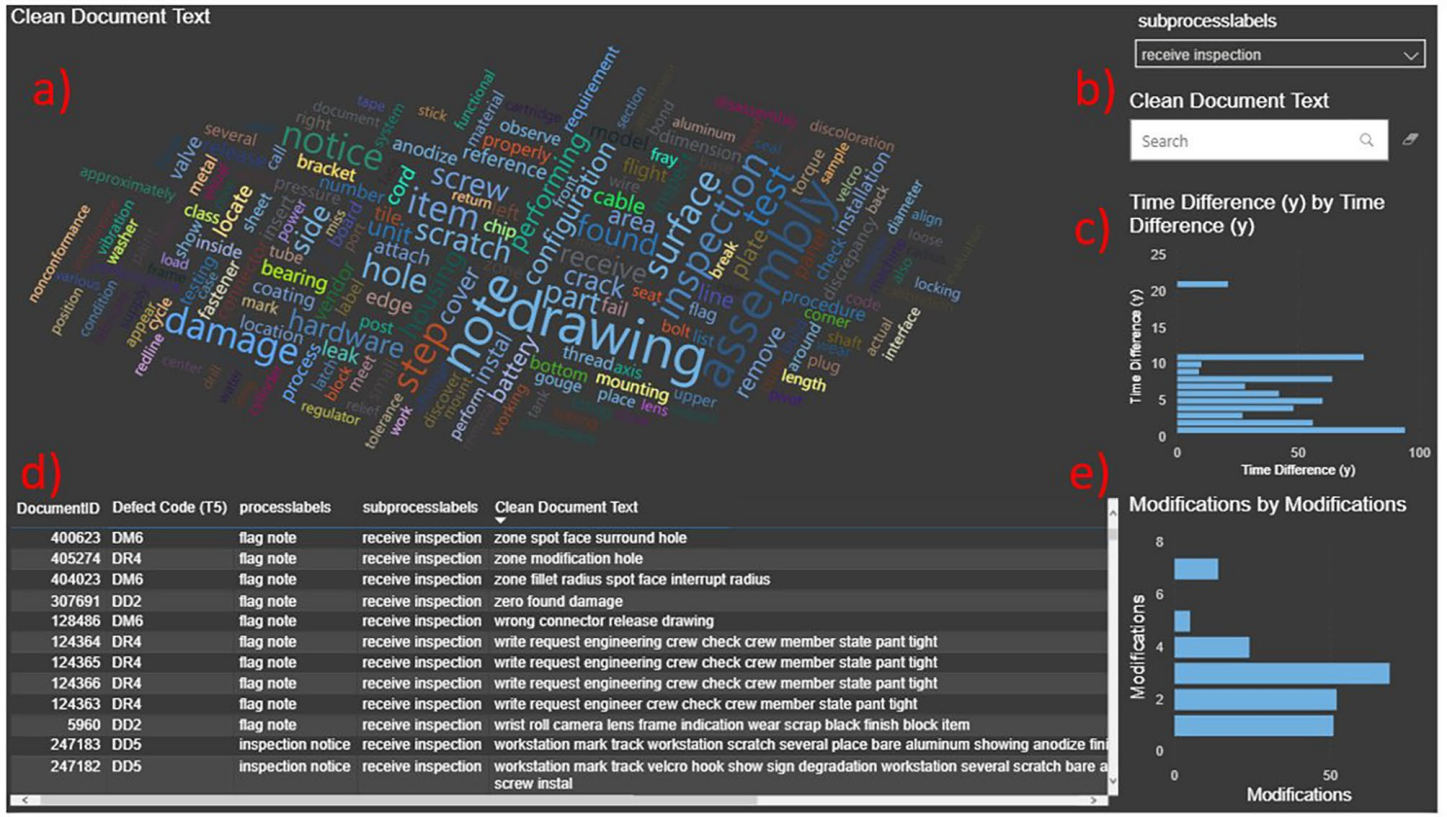
Dashboard for text analysis. (**a**) Word Map, more dominant words in the clean document text selected appear bigger. (**b**) Slicer and Query tools to select specific reports. (**c**) Time difference (time that took the report to be closed after initiation date) histogram of selected reports. (**d**) Taxonomy table containing the specific document ID, taxonomy classifications, and post processed cleaned document text. (**e**) Number of times that a report had to be modified histogram of selected documents.

Power BI was used to analyze the LDA-BERT Taxonomy results. In Fig. 2 d, a mapping table was created in order to analyze individual records clean document text, the taxonomy topics, and map them to the original discrepancy report code. At the same time, the Power BI query tool was used (Fig. 2 b) to look at specific documents within the same taxonomy subbranch or look for specific words or phrases in the document text of the records. Another Power BI capability explored was the forecasting tool (shown in Supplementary Figure S1) using Neural Networks which predict the time behavior of single taxonomy subbranches, with some hyper-parameter scanning and without need of constructing a code.

The taxonomic tree gives labels to analyze the subbranches against different data fields (Fig. 2 c, e are some examples), a key extractor can be used to plot a word map (Fig. 2 a) for a cursory understanding the text of these documents without needing to read them individually. The creation of a taxonomy helped trace a future path for association rule mining and finding casual relationships between documents.

**Fig. 3:**
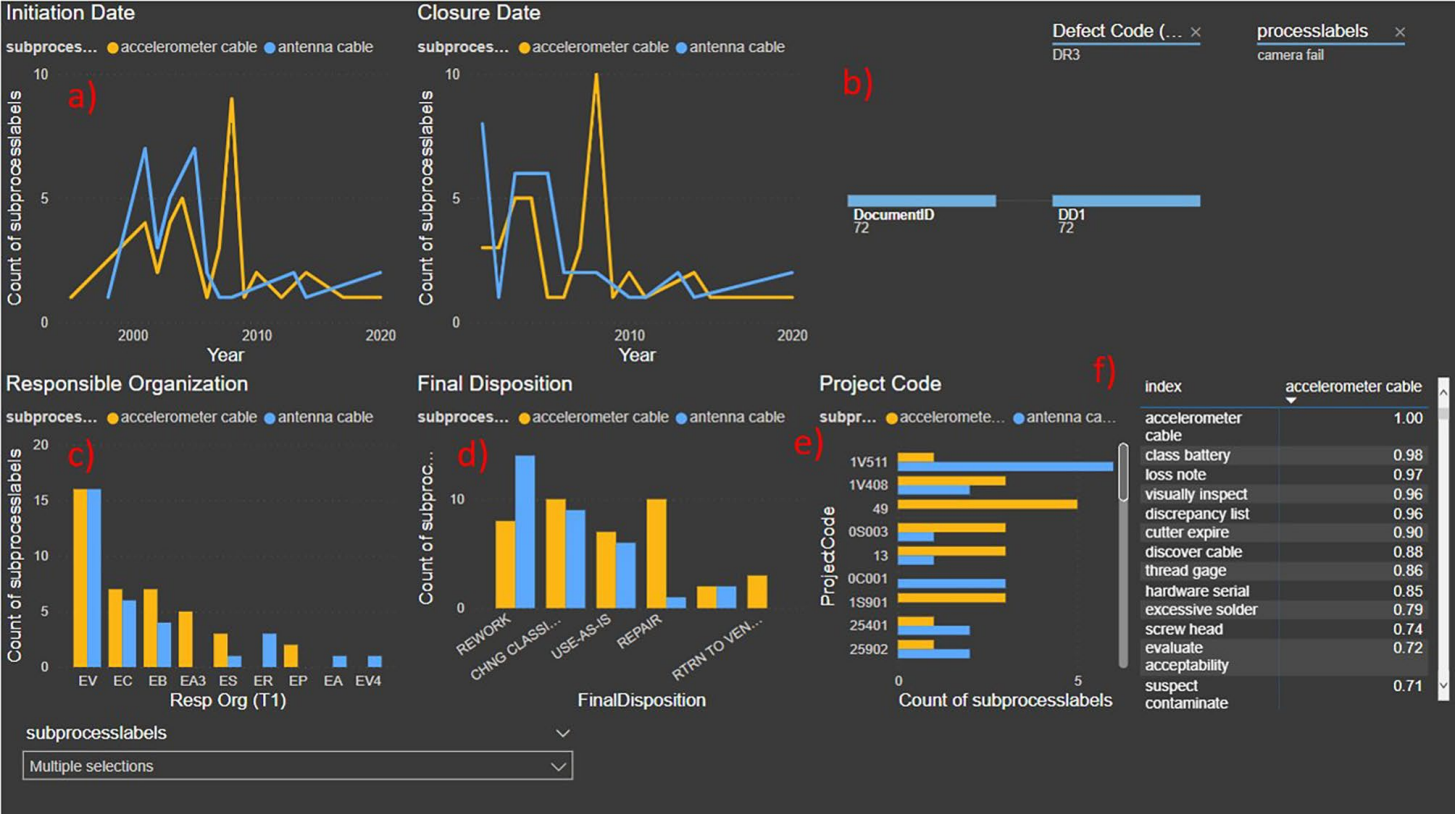
Dashboard analysis result correlations example. (**a**) Time series plot for initiation and closure dates of the selected sub-branch of the taxonomy. (**b**) Taxonomy tree to identify parent branch. (**c**) Responsible Organization histogram comparison between selected sub-branches to identify other type of correlations. (**d**) Final Disposition organization histogram comparison. (**e**) Project code histogram comparison. (**f**) Correlation coefficient matrix to identify what subbranches correlate.

Trends of each subgroups can be visualized via time series plots (Fig. 3 a). Furthermore, having subgroups makes it possible to identify correlations in between subtopics. Using other data fields from the documents (i.e. discrepancy report initiation date or closure date), a time correlation matrix between two different subgroups can be calculated. This correlation matrix can help us find specific correlations between subbranches of the taxonomy. Selecting two sub-label sets of documents that have high time correlation, it is possible to visualize other correlations in different data fields. Power BI was used to create such visualizations in Fig. 3 where correlations of between subbranches was observed across different fields like Final Disposition (3 c) and Responsible Organization (3 d).

Commercial tools like AWS Comprehend and IBM Watson Explorer were also tested to compare and validate LDA-BERT results. IBM Watson Explorer NLP have pre-built visualizations that help the user to analyze the trends, dominant phrases or words, histograms, and deviations from selected data. Watson Explorer correlation function allows the identification of different levels of correlations among groups inside the dataset. These correlations can be form by analyzing any pair of facets (variables) e.g. the user can identify relations between phrases and certain discrepancy reports. NLP from Watson Explorer identifies important part of speech as verbs, adverbs, adjectives and common phrases across the discrepancy reports. After including taxonomies and parsing rules, Watson Explorer can search through the document texts to find correlations between certain keys using context. User needs to manually explore the correlations that Watson Explorer finds to understand better the meaning of such correlations and the processes involved in the selected discrepancy reports. Watson Explorer did not find correlations for most of the demo data set of discrepancy reports leaving the user without useful results on the missing correlations groups. In order to exploit the NLP capabilities of Watson Explorer taxonomies or ontologies must be constructed. These can be built manually from an experienced user in the field, or applications developed like LDA-BERT for topic modeling and must be used to automate the creation these taxonomies. Although Watson Explorer can have a very intuitive interface, it requires some training in order to get to know all its capabilities. Also, the user needs a close contact with the administrator to construct taxonomies or make available the use of other tools. Since the text mining of the space hardware reporting documents was planned to be a one-time run, setting up IBM Watson Explorer dedicated environment and costs for longer term was not fitting. In the AWS Comprehend side, features like key phrase extraction, topic modeling, and entity recognition were tested. The key phrase extraction worked comparable to previous key extractors built early on. LDA-BERT topic modeling capabilities demonstrated to be preferred after a careful evaluation of given AWS Comprehend labels. From LDA-BERT and manual revision it was expected that AWS Comprehend would find the dominant label contributor in the reports, however AWS Comprehend failed to do so. It is speculated that AWS Comprehend topic modeling labels could have been as useful as LDA-BERT, however, that approach would have required training of custom models in AWS Comprehend bringing the testing costs and time higher without the insurance it will bring better results than LDA-BERT. Entity recognition was a feature that worked well, but the discovered entities did not contribute significantly to the information extraction.

### Cause-effect relation analysis on taxonomy branches requires explicitly written information

INDRA-Eidos extracted causal relationships from a set of records. Some of these causes repeated with different effects, and some of these effects repeat with different causes, making possible forming clusters of relationships (Suplementary Figure S2). It is worth mentioning most of the found causes and effects were meaningless (at least from a human perspective it is hard to understand the relation the model is finding between two entities). After careful revision, the list of causes and effects found were manually filtered. These interactive plots constructed using Bokeh-networkx with Python are a great way to synthesize, store, and read information. Visualizing and interacting with knowledge graphs creates a huge advantage over conventional structured text. For a validation of INDRA-Eidos results, a small subset of the DRs was selected to manually identify root cause and compared to statements found by INDRA-Eidos. There was a considerable discrepancy to what a human can find, versus the model. It is likely that the Eidos has to be modified to account for the space hardware context, or other knowledge extractor models should be tried. Using the same validation set, a Spacy matching pattern was created to try to replicate such results. This customized model was able to replicate some of the INDRA-Eidos cause and effects found. It was able also to replicate some of the validation results which were obtained after manual inspection of the documents. However, it also came with a lot of noise or non-useful relations. It is important to note that for INDRA-Eidos and Spacy both need the relation to be explicitly written on the text, models by themselves won’t be able to simulate human inference unless combined with statistical approaches or strict training.

**Fig. 4:**
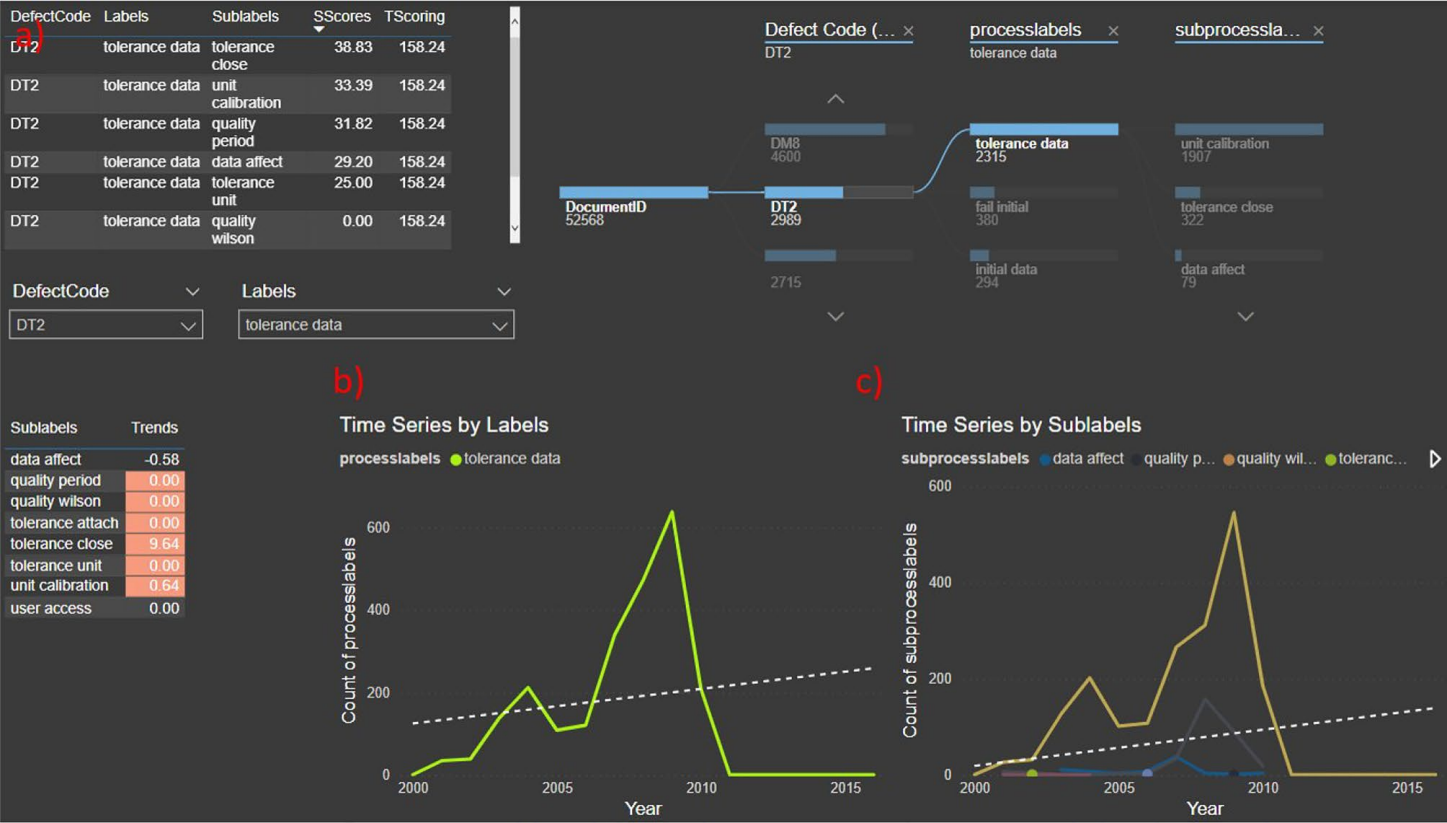
Dashboard for trending analysis. (**a**) table where single effective scoring and total effective scoring are displayed to weight the percentage of appearance of labels on their specific subgroups. The trending slopes were also computed and displayed to easily identify high trending and high scoring subgroups. (**b**) Time series with trending line by processlabels. (**c**) Time series by sublabels with aggregated trending line.

A closer look was taken to taxonomy subgroups with high scoring and high trending using a Power BI dashboard with trending metrics shown in Fig. 4. It was found that most of this DRs contain a very simple description which was not enough to describe important causes of the discrepancy or gather relevant context. The grouping itself was not perfect; however, it was always dominated by the sublabel name, and DRs within the same subgroup which were usually related to each other. NASA engineering management monitoring capabilities could be improved by having a more strict set of rules in order to close DRs, or having a field where the user can capture the likely root cause if it deemed the return on investment does not justify a fully root cause investigation.

## Discussion

A Taxonomy is a superb tool to identify key phrases and concepts as well as extract knowledge from a document text set. Correlations and trends can be found within the sub-branches showing well behaved structure. The new categorical groups may help to improve the monitoring capabilities of space hardware failure trending in the future. Interactive knowledge graphs are a useful to visualize and extract insights from a data set. There is still an area of opportunity in developing, modifying, or finding a model that can extract association rules, root cause, and knowledge from the LDA-BERT taxonomy subgroups.

In response to the reviewers’ insightful comments, we have made several revisions and additions to our manuscript. We executed Latent Dirichlet Allocation (LDA) with five topics per defect code, balancing granularity and interpretability. This structured taxonomy aids in understanding engineering process failures. We generated descriptive statistics and provided class balance details in the supplementary data file, ensuring transparency. While we acknowledge the value of comparing supervised and unsupervised models, time constraints limited this inclusion. However, we have validated LDA results through supervised model training, enhancing predictive power and ensuring meaningful, stable topics for classification. Future research could incorporate these comparisons, further strengthening our findings.

The deficiencies of INDRA-Eidos and Spacy to generate trusted root causes was a consequence of the quality of the data since most of the information had to be inferred or was not present at all in the text. The filtering of typos and special characters was also a problem since some sub-labels that were generated lacked proper context. However, after careful manual revision of the specific subgroups on the text proper context could be drawn to justify the final sub-labels. In this application the size of the data set required some automated advanced analysis, however to reap the full power of the AI ML approach would require substantive investment to train the tools to be able to extract true root cause by constructing expansive failure scenarios, but since the frequency of analysis in the future would not be to the magnitude to make this cost effective and thus we decided to use the frequency occurring labels and sublabels to establish trending as a future tool to gather the needed information to have addition insight into root cause.

The combination of diverse approaches for text mining highlighted the idea that Machine Learning is not a magical tool which will solve all your problems, but it is a logical statistical tool that performs a series of tasks. The results of these models have to again be analyzed and re-implemented several times to improve the results. These tools have their limitations but their power relies on their scalability potential in big-data settings. When the data is simple enough these tools demonstrate their true potential. However, when the data is diverse and complicated, which often occurs in real world applications, the implementation of these models scales in difficulty.

As potential future directions, we could explore the potential of open-source LLMs. This approach aligns with our need to work with private data, and hosting these models on ITAR-protected NASA servers would ensure compliance with data security requirements. Additionally, we could consider the possibility of customizing an LLM for our specific needs. Techniques such as LoRA (Low-Rank Adaptation) offer promising avenues for fine-tuning LLMs to enhance their efficiency and effectiveness in text mining applications. Such a strategy would enable us to leverage the advanced capabilities of LLMs while adhering to the necessary privacy and security constraints of our dataset.

Incorporating these advanced LLM techniques into our research represents an exciting opportunity to push the boundaries of our current findings and contribute further to the field of NLP, especially in the context of highly specialized and secure datasets.

## Methods

### Data pre-processing

In the realm of Natural Language Processing (NLP), effective preprocessing is paramount to enhance the performance of downstream tasks and mitigate the challenges posed by unstructured and noisy data. In this project, we adhere to established best practices in NLP preprocessing, incorporating techniques such as tokenization, stemming, and lemmatization. Tokenization, the process of breaking down text into smaller units or tokens, serves as the foundational step in text analysis. Stemming and lemmatization further refine this process by reducing words to their base or root forms, thereby normalizing linguistic variations. Additionally, we implement the removal of stopwords – commonly occurring words that offer little semantic value – to streamline our datasets and focus on more meaningful textual elements. This strategic approach to preprocessing not only addresses inconsistencies arising from typographical errors and user-specific language variations but also significantly augments the efficacy of subsequent NLP models in extracting and interpreting valuable insights from textual data.

### LDA as an input for BERT

**Fig. 5:**
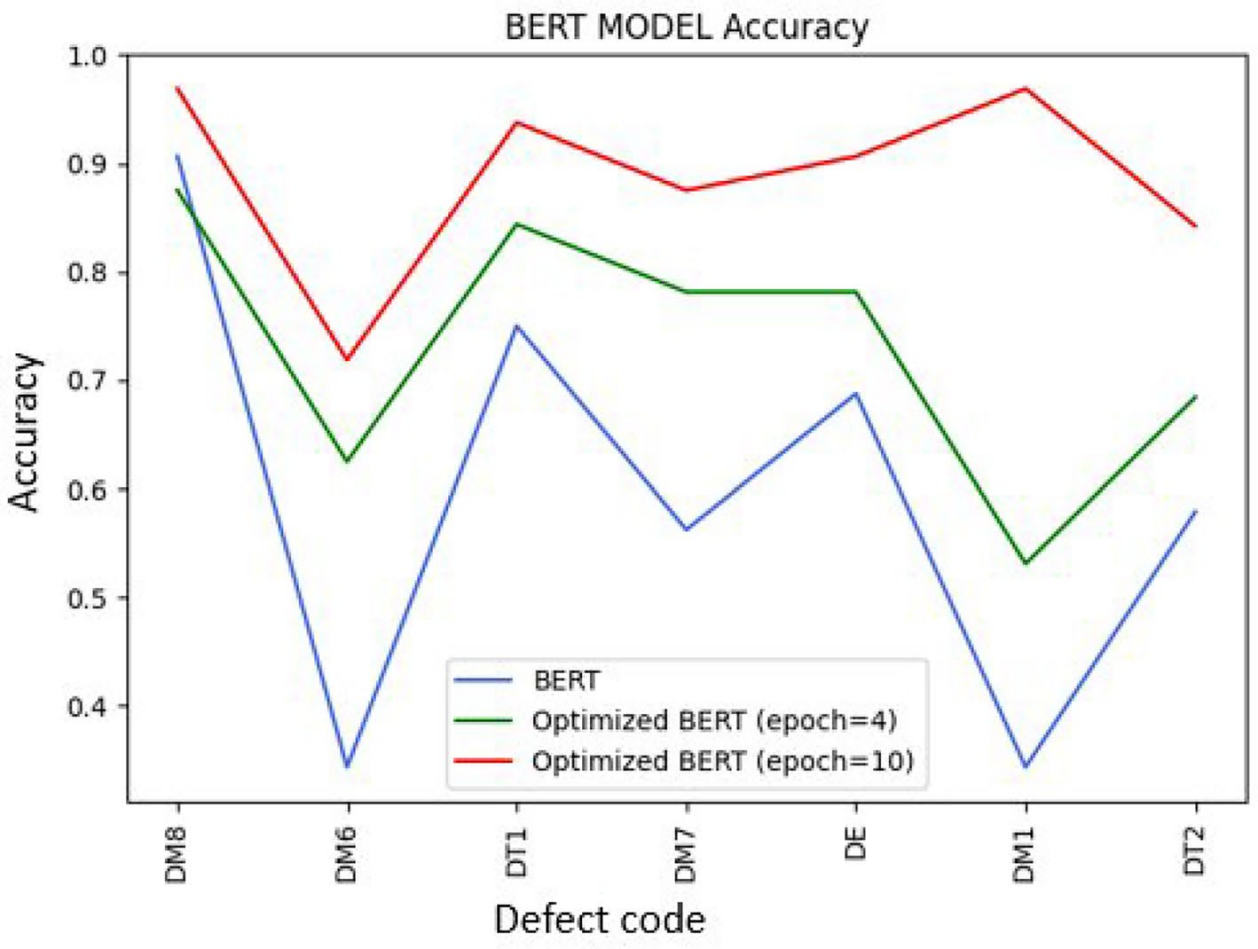
BERT Accuracy plot across different sets of Space Hardware Discrepancy Reports. The blue line shows accuracy of BERT with default parameters. The green and red line correspond to BERT with optimal parameters at different epochs (computational cycles).

One of the main disadvantages from any topic modeling technique is the number of topics for a document set is not always known. One common way to evaluate LDA performance is to evaluate the perplexity at different number of topics in order to identify the optimal number. Unfortunately, in this use case the perplexity did not decrease monotonically per batch, and could have been attributed to a source code error in the perplexity evaluation. To rule out issues with the code, a text classification supervised machine learning model was incorporated to evaluate LDA performance. We adjust BERT using an adaptive fine-tuning approach, which involves keeping the BERT weights constant and incorporating an adaptive layer for text classification. We assume LDA labels are ground truth at this step to evaluate BERT accuracy. To derive the LDA labels for use as input to BERT, we employed the following methodology: First, we ran the LDA model on our document set, where each document was represented as a mixture of topics. From the LDA output, we extracted the dominant topic for each document based on the highest topic contribution score. These dominant topics were then used as the labels for our documents, effectively translating the topic distribution into a discrete label set. This approach allowed us to map each document to a specific topic label, which was then used to train the BERT model in a supervised learning context. By treating these labels as ground truth, we could fine-tune BERT to predict the topic labels, thus evaluating its accuracy in a text classification task. As discussed in the early sections several classifications models were tested. However, BERT was chosen as main text classification model due to its high performance (benchmarks shown in Table 1). After combining the two models LDA-BERT, and performing the proper grid-searches of accuracy over the number of topics per document set, it was possible to identify the optimal number of topics by identifying the local maximums of accuracy plots. LDA-BERT Performance across several defect codes is shown in Fig. 5.

**Table 1:** Comparison of Model Performance for Text Classification task Using 5-Fold Cross-Validation. BERT Outperforms Other Models Across All Evaluation Metrics.

Model	Accuracy	Precision	Recall	F1 Score
Logistic Regression	0.8134	**0.8285**	0.8134	0.8084
Random Forest	0.7429	0.7635	0.7230	0.7313
Naive Bayes	0.7847	0.8143	0.7847	0.7659
SVM	0.7792	0.8067	0.7792	0.7713
Gradient Boosting	0.7429	0.7487	0.7429	0.7342
AdaBoost	0.4086	0.3159	0.4089	0.2963
Decision Tree	0.6487	0.6613	0.6496	0.6480
K-Nearest Neighbors	0.7072	0.7078	0.7072	0.6980
MLP Neural Network	0.7932	0.8033	0.7932	0.7897
Random Model	0.1503	0.1620	0.1503	0.1530
High Frequency Model	0.2314	0.0535	0.2314	0.0869
**BERT**	**0.8211**	0.8177	**0.8211**	**0.8149**

### Feature extraction clustering

**Fig. 6:**
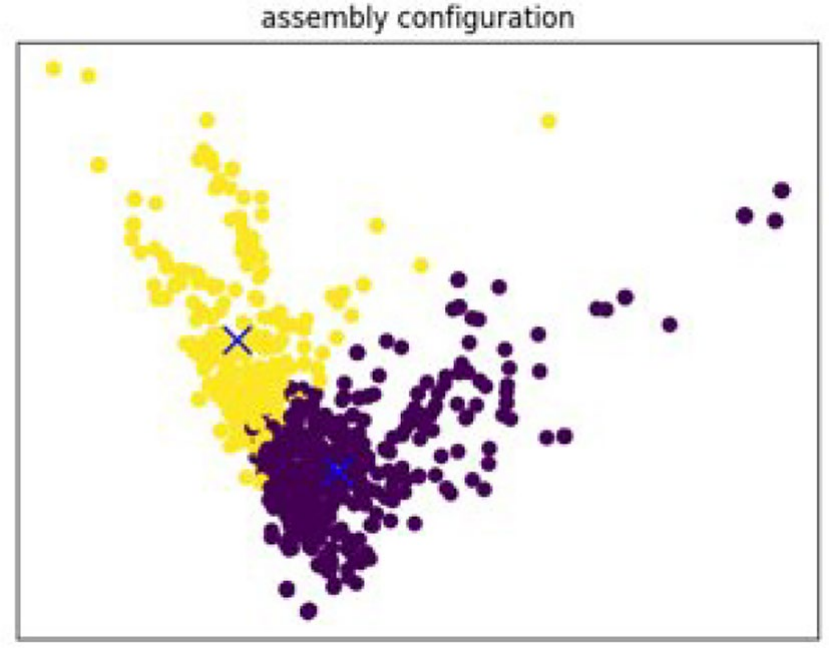
Feature Space Clustering Example. With this Unsupervised clustering technique it was possible to identify number of clusters within topics in the text data set. Each color corresponds to a distinct subgroup.

To identify the number of subtopics we used Feature Extraction Clustering^13^ instead of LDA-BERT. Although this approach can be more inaccurate than LDA-BERT (since to visualize a feature space normally a Principal Component Analysis or PCA has to be performed in order to reduce the number of dimensions of the feature space), it was found to be less computational expensive and an acceptable trade. After plotting per each topic (Fig. 6), a number of sublabels were identified from the different clusters found in the feature space. We later use this number of sublabels to further construct our taxonomy.

### Automated taxonomy

**Fig. 7:**
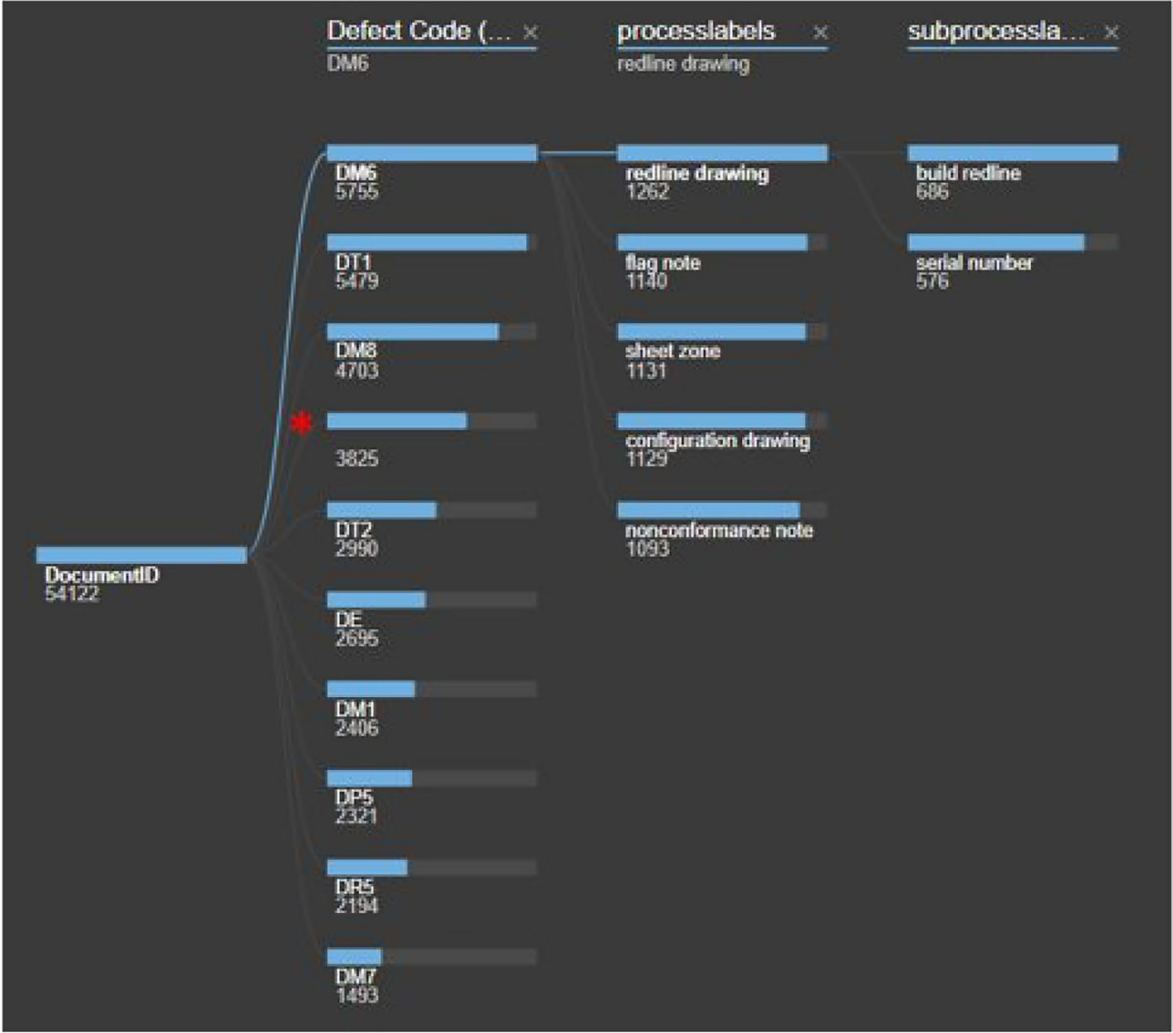
Taxonomical Tree for the classification of Space Hardware Discrepancy Reports. The first level is the defect code which is a label that already exists when filling the DRs information. Process labels correspond to the topics from LDA-BERT after running it with each respective defect code. Note it works also for DRs without defect code, which is very prominent in Open DRs. Subprocess labels is the last branch of the taxonomy and it is obtained by running LDA-BERT again on each process label.

The discovery of substructure in the topics found by feature clustering analysis (Section "Feature Extraction Clustering") hint that subgroups must be created which making possible the creation of a taxonomy for the space hardware records. With NLP the use of Taxonomies (classification trees) are key to structure text data, extract knowledge, and important concepts from the documents, and facilitate the identification of correlations and trends within the data set. Some early attempts for the automatic creation of a classification tree were made from Hierarchical clustering on a feature space^14,15^; however, due to the large number of dimensions of a feature space this approach was challenging to implement. Additionally, the dendrograms cut off and the labeling of the numerous branches turned out to be arbitrary which added additional trouble to this approach. A more direct approach was taken by creating a basic taxonomy by running LDA-BERT on the process labels instead of the defect codes to form subgroups (sublabels). An example of the obtained taxonomy is shown in Fig. 7. Some of the sublabels of this taxonomy were mapped to old failure codes used by NASA in the past, in order to become more informative and delete ambiguity.

#### Improving LDA-BERT taxonomy labels

**Fig. 8:**
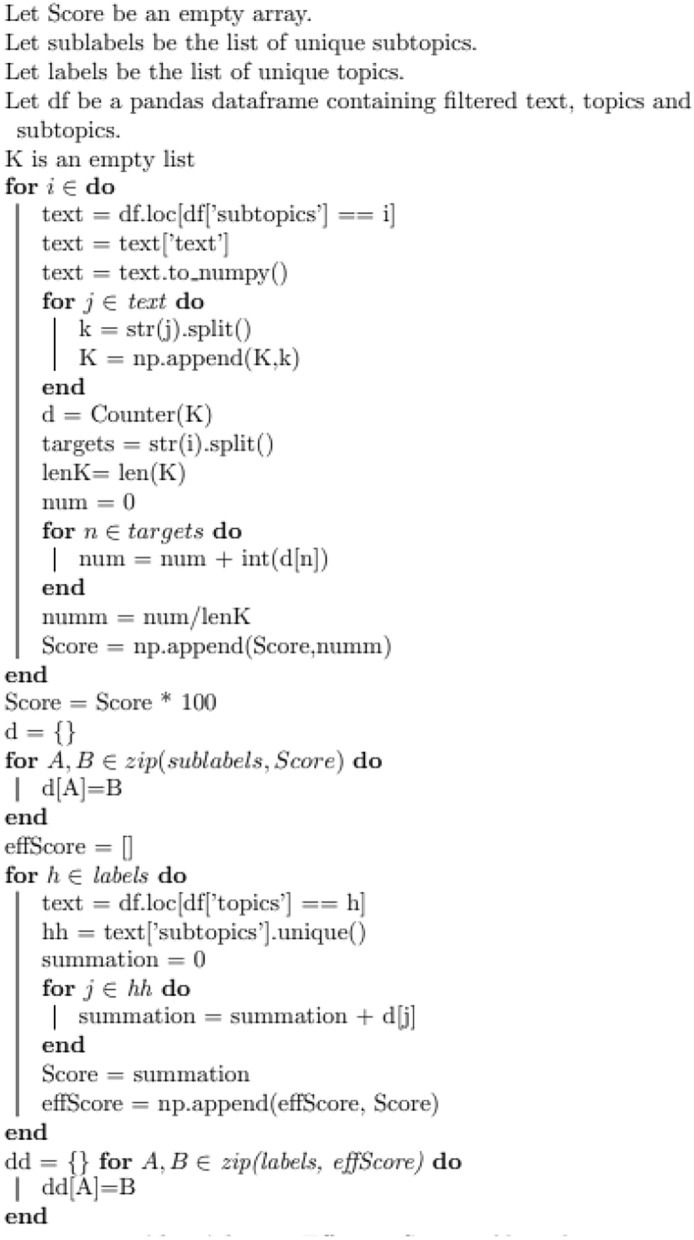
Implementation of Effective Scoring Algorithm. This algorithm was created to quantify the quality of the taxonomic labels without the need of user input. Generally, this algorithm reads through the group of records with same labels and calculates the percentage of appearance of the specific words forming the labels in the corpus of the records.

The resulting topics and subtopics from the LDA-BERT taxonomy create groups of records that follow similar trending across different variables (e.g. Closure Date, Initiation Date, Final Disposition, Responsible Organization). However, a natural question emerges: Do the words from these topics appear in the actual records? There is a need to evaluate the quality of these labels. In the ideal scenario, a human would read through each group of records and evaluate if most of the records are accordingly grouped together i.e. share common characteristics or talk about the same space hardware engineering process. However, it is unpractical to perform such a task due to the large size of the data set being analyzed. In order to evaluate quantitatively the resulting topics and subtopics, an effective scoring algorithm was developed. This algorithm would read through the text of the records and calculate the percentage of appearance of the subtopic and topic words on their respective corpus. An implementation of the algorithm outline is shown in figure (Fig. 8). Then, a threshold is considered to identify lower percentage of appearance for specific topics. New grid-searches over the number of low performance topics were created. Naturally, the effective scoring increases as the number of subtopics within each topic is increased. This occurs since LDA-BERT was constructed such that subtopics name will not repeat, eventually with a sufficient large number of subtopics will cover the entire corpus. To get rid of this dependency the highest increasing rate of performance was considered to identify the ideal number of labels. Furthermore, the subtopic labels were manually revised, and to make them more informative and easier to use, some were modified using a key extractor over the specific subgroup of records.

While manually revising the subtopic labels, a key extraction within the respective subgroups was not always sufficient to determine an ideal label. A more basic statistical approach was considered using Markov chains. Markov Chains are used in mathematical systems to express transitions from different states based on probability rules. In NLP, these states are words, and the probabilities are the weighted expectation of finding the next word. A Markov dictionary was constructed where a word is followed by a ’next word’ which is found in the given subgroup text. The most likely next word is selected, and the algorithm is repeated to form any desired title length. The different branches can be visualized in interactive tree diagrams (Supplementary Figure S3).

### Beyond topic modeling and text classification: casual relationships extraction from text data with INDRA-Eidos and Spacy rule matching

A taxonomy yielded in important results for correlations and trends within specific subgroups of the records. However, there was still a knowledge gap to identify the root causes of the discrepancy reports in an automatic way. To overcome this challenge, a set of association mining models were tested (apriori algorithm see section "Beyond topic modeling and text classification: casual relationships extraction from text data withINDRA-Eidos and Spacy rule matching") and INDRA-Eidos^16^. The Integrated Network and Dynamical Reasoning Assembler (INDRA) is an information assembler that extracts statements from text in molecular biological systems. These statements can be events, influences (cause-effect), annotations, parameters, monomers, and rules. INDRA can also be used to predictive and explanatory models by elaborating in extracted statements. Eidos is built on INDRA, and its main application is to extract statements from non-molecular biological systems. INDRA-Eidos can be run with a Python-Java bridge or webapp application from Scala. The model was later run in Linux by hosting an Eidos server in via linux terminal window, and a virtual Python environment Jupyter notebook which called the server in another terminal. This model was used to extract cause and effect relationships from the text of records. By manually inspecting the causes and effects, since few were produced compared to the number of records fed to the model, non-fitting relationships were removed. Then, the list of causes and effects were visualized using Bokeh-networkx Interactive Knowledge Graphs^17,18^ (Suplementary Figure S2). Interactive knowledge graphs are useful to form clusters of common causes and effects that INDRA-Eidos or Spacy are able to find.

It is not possible to customize the rules that INDRA-Eidos uses to find cause and effect relationships without modifying the source code. Spacy rule matching^19^ can be used to build a series of dependency grammatical patterns. A visualization of this is displayed in Suplementary Figure S4. After careful experimentation and grammatical analysis a general rule pattern matching was created to emulate manual inspection of the documents. Interactive Knowledge Graphs were also used to visualize Spacy’s results.

#### Early attempts for root cause and association rule extraction: factor analysis and apriori algorithm

Before attempting INDRA-Eidos, other approaches were tested. One approach is Fast dimensional analysis for root cause investigation^20^. There are several tools for dimensional reduction analysis, one of them is Factor Analysis which is an unsupervised machine learning algorithm for dimensional reduction. The idea is to group similar terms with certain commonalities (or similar variances) found in the records. These groups should be related to each other and come from a common root cause. An additional use case of Factor Analysis is to be used as an exploratory method. This can be used to identify hidden factors that influenced the set of recorded variables, which in turn helps reduce the number of variables in a data set. In NLP, word matrices and feature spaces have very large dimensions. The idea was to use Factor Analysis to identify key features in a document text subset of data from a similarity matrix. However, the Bartlett’s test of sphericity^21^ and Kaiser-Meyer-Olkin (KMO) Test^22^ resulted with inconclusive scorings for the hardware failure reports data set. Another attempt to extract relationships and association rules from the hardware records was via Apriori Alogrithm which has been applied for association rule mining before^23^. Since it was designed for transaction type data structures it did not perform as expected. Some data rearrangement was attempted with with taxonomical LDA-BERT grouping and time series consideration, however, results were inconclusive.

### Other topic modeling and text classification tools tested

#### Topic modeling alternatives: customized topic modeling with BERTopic and TF-IDF Key extractor

**Fig. 9:**
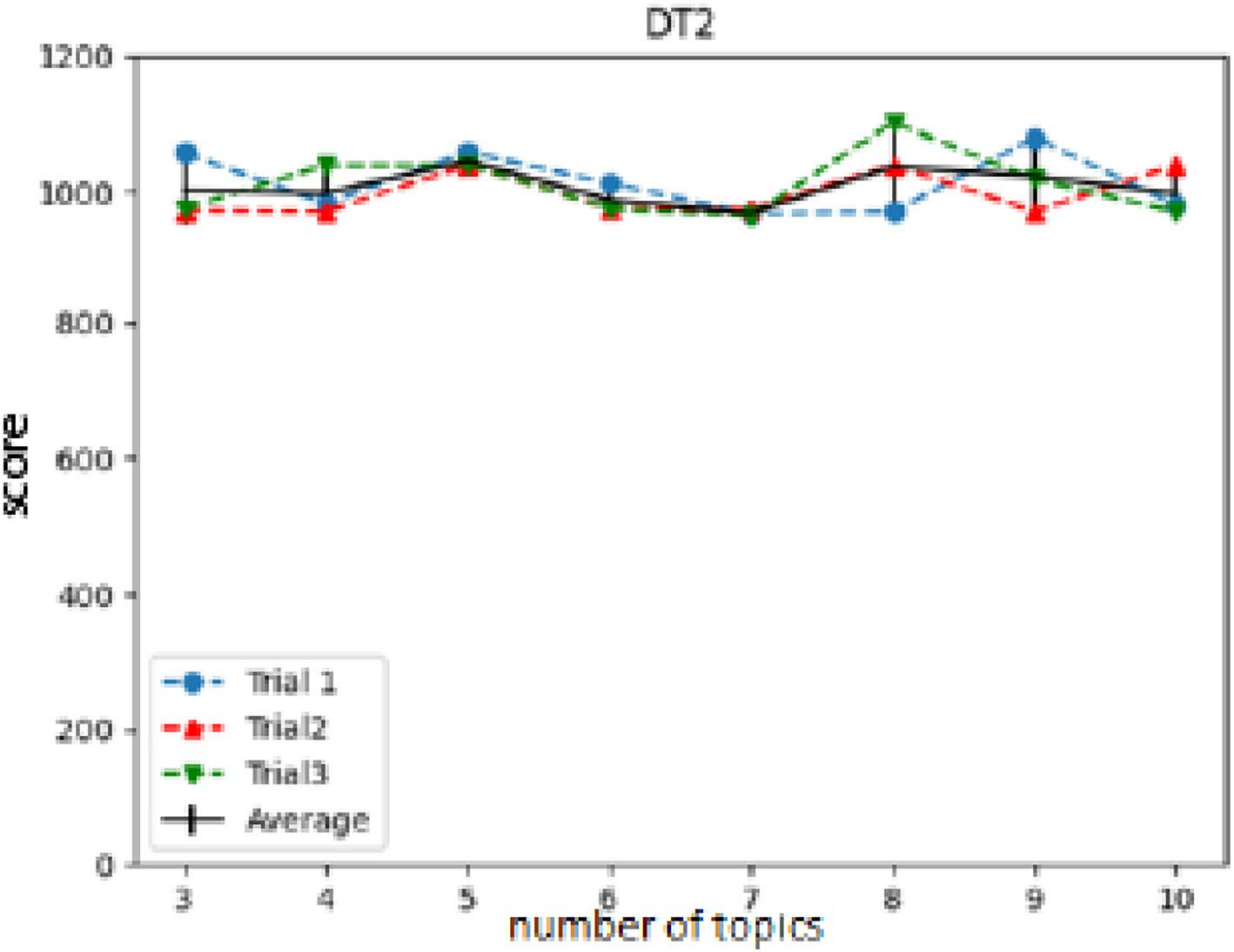
BERTopic gridsearch scanning different number of topics, located in the x-axis, within a defect code (DT2 in this example). The vertical axis corresponds to a scoring provided by BERTopic, note that similar scores are given to the scanned numbers of topics, this is the reason why it was not possible to identify an optimal number of labels using this model.

BERTopic^24^ is a topic modeling technique which is different from LDA in the use of transformers and class-based TF-IDF analysis. BERTopic modeling creates clusters from a set of document text and assigns topics to the most dominant words. After computing a rating grid-search over the possible number of labels, the model had a monotone behavior for most of the defect codes (Fig. 9). Also, the model had large variance relative to the peaks, it was not possible to identify the number of processes labels within a DR defect code. BERTopic Topic Modeling tool was ultimately pursued since it was demonstrated to have a poor performance in the data set compared to LDA. An example of a grid-search is shown in Fig. 9. The TF-IDF analysis for a text data set is well known^11^. A key extractor can be built by weighting the TF-IDF score of each word (or group of words e.g. bigrams) and selecting the highest scores from a corpus. The extracted key phrases can be used as topics or labels. Then, these labels can be assigned to each document by scanning through it, and identifying if it contains the desired topic. However, this method ignores the overlapping of topics within the same document and in turn does not perform well with documents which can contain multiple topics as is the case in real-world applications where hardware discrepancies and failures have multiple issues or events which occur to create said discrepant outcome.

In light of the insightful feedback provided by the reviewers, we recognize that our exploration of the BERTopic model’s capabilities may not have delved into its full potential, particularly in terms of parameter optimization and customization for our specific use case. BERTopic, distinct from traditional approaches like LDA, offers a rich set of configurable options, notably in its clustering phase where HDBSCAN is utilized. This aspect of the model presents a significant opportunity for fine-tuning to achieve more nuanced and case-specific results.

For future iterations of this research, we propose a more thorough investigation into the parameter space of BERTopic, with a focus on settings that facilitate smaller cluster sizes. This approach is expected to yield a larger number of compact, well-defined topics that could be more closely aligned with the intricacies of our dataset. Additionally, exploring the model’s capabilities in soft clustering, allowing documents to associate with multiple topics, presents another promising avenue. This could be complemented by utilizing either a sophisticated Language Model or expert intervention for a more refined and comprehensive description of the topics.

Such an enhanced approach would not only address the current limitations but also unlock the full potential of BERTopic in providing a deeper, more accurate analysis of textual data. By embracing these modifications, we aim to significantly refine our model’s performance and contribute further to the field of topic modeling in NLP.

#### Multi-class logistic regression and multinomial naive bayes

Before testing BERT, common classification tools in machine learning were considered as an starting point such as multi-class logistic regression and multinomial Naive Bayes. Classically, a Binomial Naive Bayes classifier contains binary features to classify with two different labels; however, when dealing with more than two types of classification its necessary to use a Multinomial approach. It is important to note the Multinomial algorithm assumes discrete features, which is contrary to LDA (considers only the overlap of features within the same document) and is a considerably better representation of space hardware discrepancy reporting. Multi-class Logistic Regression is one of the most basic machine learning classification models. One of the main advantages of this model is the extensive number of solvers it contains, making it one of the most versatile classification models since many of these algorithms can be tested without excessive complications. After tuning hyperparameters manually, topic modeling and text classification combinations, the classifiers showed a variety of accuracies. No model was able to have a reasonable stability across different defect codes i.e. their accuracies had high variability. We opted to consider more complex multi-class classification techniques.

#### CNN and RNN for text classifications

After trying Naïve Bayes Classifiers in the intent of finding appropriate text classification techniques, some deep learning neural networks with basic architecture were used. The neural networks tested were Convolutional Neural Networks (CNN) and Recurrent Neural Networks (RNN) for text classification. Convolutional Neural Networks are popular for their high performance in image, speech, and audio recognition. The idea of using a CNN as a text classifier is not new^25^. After transforming a document text to vectors or matrices with word embedding, it is relatively easy to play with different architectures (different number of layers and nodes) and incorporate common techniques such as padding and pooling.

Although more efficient and elaborate RNN have been created^26^, in this project only basic structures were tested. Building neural networks from scratch along with training can become computational expensive (even more than BERT, since BERT already is pre-trained). Normally, large neural network configurations and excessive training leads to over-fitting which decreases the performance to correctly classify record text inputs which have not been trained to recognize. Considering only small rudimentary neural network architectures was not sufficient to yield in satisfactory results, however creating a complicated architecture and training it would have taken a long computational time. For this reason, BERT which is already a pre-trained language model was utilized. Pre-optimization and post-optimization, BERT demonstrated higher accuracies across different defect codes, outperforming Naïve Bayes Classifiers and CNN, RNN text classification approaches.

#### Other natural language processing techniques

This project was not limited to topic modeling and text classification with LDA and BERT, but was extended to commercial tools like Amazon Web Services (AWS) Comprehend and IBM NLP Watson Explorer. A diverse extensive list of models were also tested. Some were previously mentioned in the earlier sections, others include: ASER^27^, graph databases with Neo4J, neural topic modeling with knowledge distillation^28^, hierarchical topic modeling with corextopic^29^, a collection of summarization models^30^, and NLP Cloud partnered with NVIDIA. Most of these open source models were hard to implement because of code deprecation. After extensive work in modifying the source code for some of these models results were still inconclusive or not as desirable as other models used previously. On the other hand, corextopic was easy to implement, however to provide seeded topics they must be in the text for the model to populate around it. Since the desired seeded topics were not specifically in the text, or the infrequency of occurrence was not consistent, it did not match our use case.

## Supplementary Information

Supplementary Information 1.

Supplementary Information 2.

## Data Availability

The datasets generated and/or analysed during the current study are not publicly available due to containing sensitive documents with NASA’s engineering processes information but are available from the corresponding author on reasonable request.
