# Supplementary material for: Taxonomical modeling and classification in space hardware failure reporting
Source: Sci Rep. 2026 Jan 21;16:5868. doi: 10.1038/s41598-026-36813-7 (PMC12894956; doi:10.1038/s41598-026-36813-7)
Supplement: Supplementary file 2 — Supplementary Information 2. [file 41598_2026_36813_MOESM2_ESM.pdf]

Supplementary Figures

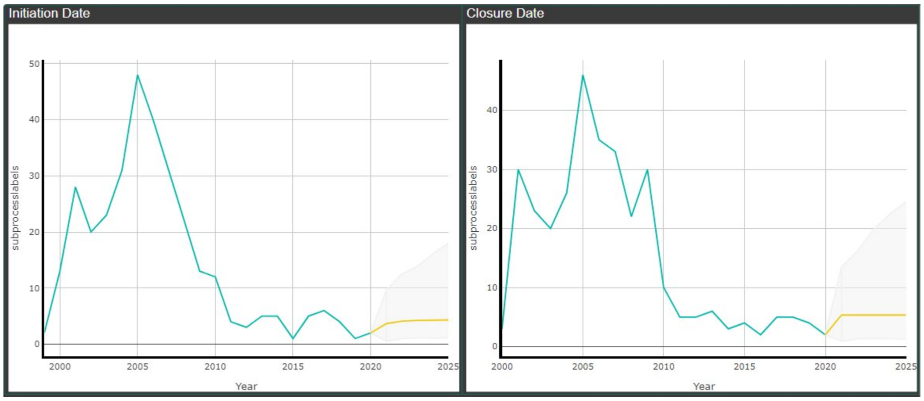

Suplementary Figure S1. Forecast using Neural Network by MAQ Software on a subbranch group from LDA-BERT Taxonomy. Green section corresponds to the reports time series data, while the yellow section corresponds to the predicted trend for the future.

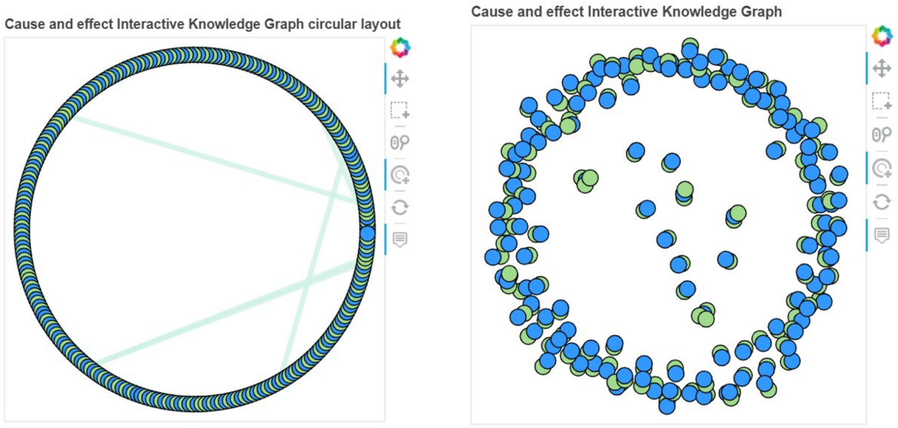

Suplementary Figure S2. Interactive Knowledge Graph created with Bokeh-networkx from INDRA-Eidos results. Blue nodes corresponds to effects, green nodes correspond to causes. Icons in the right correspond to interactive tools like pan tool, selection box, zoom wheel, and reset. Some basic interactive tools were included like wheel zoom, highlighting tool, and hover display properties.

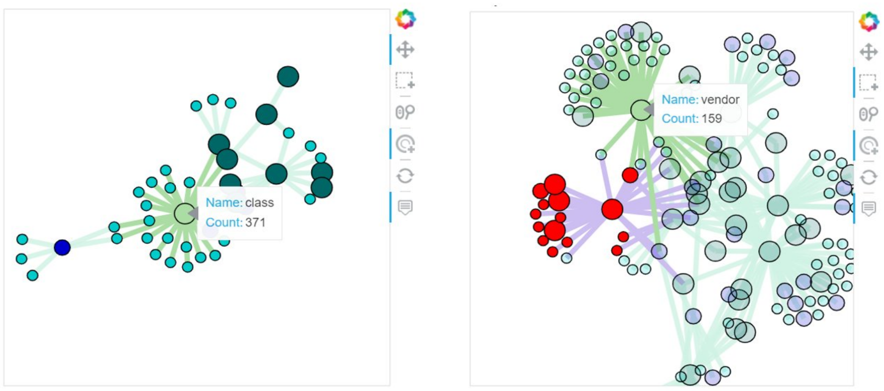

Suplementary Figure S3. Markov Chain Tree Interactive Graphs examples from specific subgroups from LDA-BERT Taxonomy. Larger dark green nodes correspond to higher word count, medium blue nodes correspond to 10-25 word count, and smaller cyan nodes correspond to below 10 word count. Purple edges and orange nodes are highlighted from selection box tool.

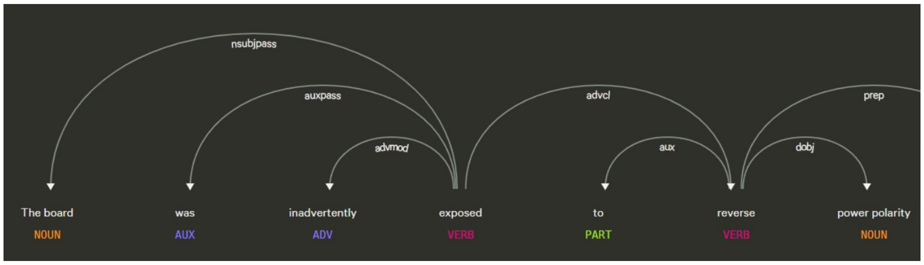

Suplementary Figure S4. Spacy dependency rules visualization. Spacy automatically labels the provided text with linguistic tags (NOUN, ADV, VERB, etc...) shown under each word. It also creates gramatical relations between words which are shown on top with arrows. This image was generated by using Spacy's interactive tool. After visualizing several sentences, patterns can be identified to have specific relationships being extracted to match an specific use case.

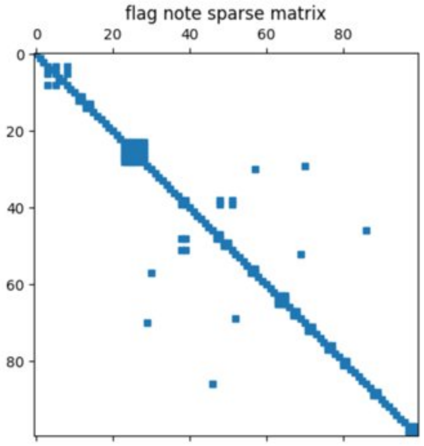

Suplementary Figure S5. Sparse Similarity Matrix Example. Indexes pairs correspond to the documents being compared, data points represent similarity larger than the provided threshold. Note only the first 100 documents with flag note process labels are shown in this figure.
